# In-Bed Posture Classification Using Deep Neural Network

**DOI:** 10.3390/s23052430

**Published:** 2023-02-22

**Authors:** Lindsay Stern, Atena Roshan Fekr

**Affiliations:** 1 Institute of Biomedical Engineering, University of Toronto, Toronto, ON M5S 3G9, Canada; 2 KITE Research Institute, Toronto Rehabilitation Institute, University Health Network, Toronto, ON M5G 2C4, Canada

**Keywords:** postures, classification, deep learning, pressure ulcer

## Abstract

In-bed posture monitoring has become a prevalent area of research to help minimize the risk of pressure sore development and to increase sleep quality. This paper proposed 2D and 3D Convolutional Neural Networks, which are trained on images and videos of an open-access dataset consisting of 13 subjects’ body heat maps captured from a pressure mat in 17 positions, respectively. The main goal of this paper is to detect the three main body positions: supine, left, and right. We compare the use of image and video data through 2D and 3D models in our classification. Since the dataset was imbalanced, three strategies were evaluated, i.e., down sampling, over sampling, and class weights. The best 3D model achieved accuracies of 98.90 ± 1.05% and 97.80 ± 2.14% for 5-fold and leave-one-subject-out (LOSO) cross validations, respectively. To compare the 3D model with 2D, four pre-trained 2D models were evaluated, where the best-performing model was the ResNet-18 with accuracies of 99.97 ± 0.03% for 5-fold and 99.62 ± 0.37% for LOSO. The proposed 2D and 3D models provided promising results for in-bed posture recognition and can be used in the future to further distinguish postures into more detailed subclasses. The outcome of this study can be used to remind caregivers at hospitals and long-term care facilitiesto reposition their patients if they do not reposition themselves naturally to prevent pressure ulcers. In addition, the evaluation of body postures and movements during sleep can help caregivers understand sleep quality.

## 1. Introduction

In-bed posture monitoring has become an area of interest for many researchers to help minimize the risk of pressure sore development and to increase sleep quality. Pressure sores can develop when there is constant pressure applied to a region of the body, resulting in a lack of blood flow to this region and the potential of injury to surrounding skin and/or tissue as time continues. As of 2020, approximately 60,000 people die worldwide each year due to pressure sores [1]. Those with pressure sores are 4.5 times more at risk of death than those with similar health factors but no pressure sores [1]. The caregivers and hospital staff are often tasked with monitoring patients with pressure sores, which can be a laborious task and can be taxing on hospital resources [2].

On the other hand, sleep quality can also be determined by evaluating body postures and movement during sleep [3,4,5]. Good sleep quality is beneficial to the health of a person as it can improve work efficiency, strengthen the immune system, and help maintain one’s physical health [6]. However, poor sleep quality can be very harmful to people as it can cause extreme fatigue and emotional exhaustion, and increase the risk of cardiovascular diseases as well as obesity and diabetes [6,7].

Currently, in-bed posture monitoring consists of using either an infrared video camera or wearable devices. However, there are some limitations to the use of these devices. For example, infrared video cameras are highly sensitive to environmental changes, such as blanket movement, and have privacy concerns. Wearable devices, such as rings and wristbands, can be obtrusive to sleep, reducing a person’s sleep quality, and are susceptible to motion artifacts [6]. Therefore, an unobtrusive privacy-preserving contactless system that can remind caregivers to change the patient’s body position frequently is needed. The use of smart mats has been an area of investigation to monitor people in and outside of hospitals to reduce the strain on caregivers, eliminate privacy concerns, and increase the accuracy of in-bed posture detection.

There are a variety of studies that have investigated in-bed posture detection using a smart mat composed of either pressure or force sensors. Many of these studies either classified the supine and prone positions as one class or ignored the prone position entirely, as these two positions can be difficult to distinguish. Additionally, these studies either examine their subjects in a simulated environment, where the investigator instructs the subject to lie in different positions, or in a clinical environment, where the subjects can lie in any position, and the investigation is typically performed overnight. Poyuan et al. conducted a simulated study on 13 participants with three sleeping postures (supine, right, and left). This study used a mat with 1048 pressure sensors and used a deep neural network to classify each position. They could achieve an accuracy of 82.70% [8] using 10-fold cross validation. Similarly, Ostadabbas et al. conducted a simulated study on nine subjects with three sleeping postures: supine, right, and left. This study used a heat map image of a mat with 1728 resistive sensors to train a k-nearest neighbor algorithm. They achieved an overall accuracy of 98.4% using hold-out cross validation [9]. This study only reported the accuracy values and did not mention any details about the amount of data they used, especially if the dataset was imbalanced. Metis et al. conducted a simulated study on three subjects with three sleeping postures. This study used a mat with 1024 pressure sensors and used principal component analysis and a Hidden Markov Model to classify these three postures, resulting in an accuracy of 90.40% using 10-fold cross validation [10]. These previous studies did not evaluate their models by leave-one-subject-out (LOSO) validation which is necessary to yield unbiased performance estimates of the models. This is a very important factor in the body position monitoring application since the final model is supposed to be used with new users. In addition, the previous literature on different applications showed that most of the time, the accuracy value drops when using LOSO compared to 10-fold and hold-out validation [11,12].

In this study, we aim to classify heat map videos of three main in-bed postures (supine, left, and right) using a 3D Convolution Neural Network (CNN) model, and compare them to image classification results using four 2D CNN models. We will evaluate our models based on both 5-fold and LOSO cross validations after addressing the class imbalance to compensate for fewer data points in the minority groups.

## 2. Materials and Methods

### 2.1. Dataset

The data used in this paper were collected by Pouyan et al. [8,13]. To our knowledge, this is the first and only open-access dataset available for in-bed posture classification. This dataset was collected in 2017 and consisted of two parts: the first part was collected on the Vista Medical FSA SoftFlex 2048 pressure mat located on a regular mattress, and the second dataset was collected on the Vista Medical BodiTrak BT3510 pressure mat on a sponge and air mattress [13]. Pouyan et al. used 2D deep learning model for subject identification in different body positions. They reported an overall accuracy of 82.73% (85.5% for supine positions, 80.4% for right-side positions, and 82.3% for left-side positions) using 10-fold cross validation [8].

**Figure 1:**
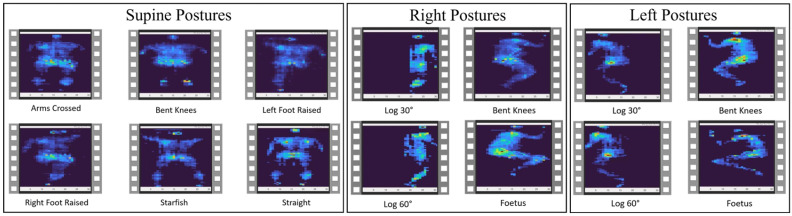
Sample images of the different postures for supine, right-side, and left-side positions.

In this paper, only the first portion of the dataset is used to classify body postures rather than subjects. The pressure data were collected with a sampling rate of 1.7 Hz. These data were reconfigured as heat map images and video files for each posture and subject. Thirteen subjects were evaluated in this dataset, each lying in 17 different postures. Out of these 17 postures, 4 are sleeping on the right side, 4 on the left side, and 9 are sleeping in the supine position. A total of 14 of these sleeping postures were recorded on a flat bed (zero incline), whereas three of the supine positions were recorded at different bed inclines, which are excluded from our analysis to ensure consistency within the data. Therefore, in total, there were 182 sleeping postures: 52 right-side, 52 left-side, and 78 supine postures. Figure 1 displays image samples of each posture.

### 2.2. Data Preprocessing

The pressure data were initially provided in text files where each row corresponded to a frame of data. These data were reconfigured into a heat map image, and then all images were merged to construct a single video file. All extracted videos were compressed into 16 frames (9 s videos) with a size of 112 × 112 pixels.

### 2.3. Inflated 3D Model

The Inflated 3D (I3D) model is a pre-trained two-stream Convolution Neural Network (CNN) video classifier created by Carreira and Zisserman [14]. This classifier was pre-trained on the Kinetics-400 dataset, which includes 400 human actions. This classifier uses two subnetworks: a video network, which is trained with RGB data, and an optical flow network, which is trained with optical flow data [14]. We evaluated our models based on two cross validation techniques: 5-fold and LOSO.

#### 2.3.1. Hyper-Parameter Tuning

For the I3D model there were four main hyper-parameters used in training and validation: batch size, number of iterations, number of epochs, and learning rate. We tried three different batch sizes: 12, 14, and 20; iterations: 30, 60, and 120; epochs: 10, 50, 100; and learning rates: 0.1, 0.01, and 0.001 to find the best model with both 5-fold and LOSO cross validation.

#### 2.3.2. Imbalanced Dataset

The dataset used in this paper was imbalanced, meaning that there were more videos of supine postures than right and left sides. To overcome this challenge, each cross validation method was evaluated in three ways: down sampling, over sampling, and class weights. Down sampling occurs when you down sample the majority class (supine class in our case) to meet the number of samples in the minority class (left and right sides). Therefore, 52 videos were randomly selected from the supine data so that all classes contained the same number of videos to train the model with a balanced dataset. This procedure was repeated five times to find the average performance of the model.

In contrast, over sampling does the opposite. Over sampling occurs when you repeat random samples from the minority classes to meet the number of samples in the majority class. In our dataset, 26 videos were randomly duplicated in the right and left-side classes so that all classes are balanced out with 78 videos. This procedure was also repeated five times to find the average performance of the model.

Since under sampling may cause under fitting due to lack of data and over sampling may result in over fitting and poor generalization of the model, a third method called ‘class weight’ was also used. This method assigns a weight to each class so that the class with more samples will have a lower weight than the minority class, meaning that the I3D model can learn equally from all classes and will not be biased to the class with more samples which in our case is the supine class. The weights for each class were determined using Equation (1).

(1) $$weight(class(x))=\frac{total\;number\;of\;videos\;\left(images\right)}{total\;number\;of\;classes*number\;of\;videos\;\left(images\right)\;in\;class\;x}\;\;$$

### 2.4. Pre-Trained 2D CNN Models

Four pre-trained 2D CNN models were used to compare the results with the I3D model. The models evaluated were AlexNet, ResNet-18, GoogleNet, and ShuffleNet. All four models were pre-trained on the ImageNet database, composed of over a million images and 1000 object categories. AlexNet is 8 layers deep, ResNet-18 is 18 layers deep, GoogleNet is 22 layers deep, and ShuffleNet is 50 layers deep [15,16,17,18]. Transfer learning was used within each of these models to apply the architect of the algorithm to in-bed posture classification model. These four models were evaluated using both 5-fold and LOSO, similar to the I3D model. Additionally, the same hyper-parameter tuning and technique for imbalanced datasets were used.

## 3. Results

The classification metrics, such as accuracy, sensitivity, specificity, and F1-Score, shown in Equations (2)–(4), of different models with different hyper-parameters were calculated, and the best models for LOSO and 5-fold cross validations were selected.

(2) $$Accuracy=\frac{T_{p}+T_{n}}{T_{n}+T_{p}+F_{n}+F_{p}}\;\;\;\;\;\;Sensitivity=\frac{T_{p}\;}{T_{p}+F_{n}}$$

(3) $$Specificty=\frac{T_{n}}{T_{n}+F_{p}\;}\;\;\;\;\;\;\;\;\;\;\;\;\;Percision=\frac{T_{p}}{T_{p}+F_{p}}$$

(4) $$F1-Score=2*\frac{Percision*Sensitivity}{Percision+Sensitivity}$$

**Figure 2:**
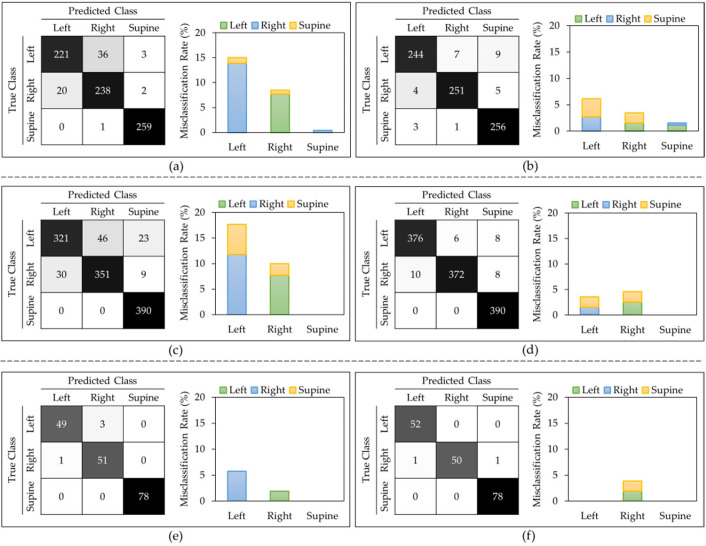
I3D model: Confusion matrix and misclassification rates of all 5 down-sampling runs for (**a**) LOSO and (**b**) 5-fold. Confusion matrix and misclassification rates of all 5 over-sampling runs for (**c**) LOSO and (**d**) 5-fold. Confusion matrix and misclassification rates of class weights model for (**e**) LOSO and (**f**) 5-fold.

*T_P_*, *T_N_*, *F_P_*, and *F_N_* denote true positives, true negatives, false positives, and false negatives, respectively. The epoch number of 10, iteration of 60 for 5-fold and 120 for the LOSO, with a learning rate of 0.001 are achieved during hyper-parameter tuning. Table 1 displays the classification metrics for each method for the I3D model. Figure 2 shows the Confusion Matrices and Misclassification Rates for all five down-sampling repetitions (52 videos per class), for all five over-sampling repetitions (78 videos per class), and for the class weight model (54 videos per right-side and left-side classes, 78 videos per supine class) with both LOSO and 5-fold cross validation. From the three approaches that were evaluated for the I3D model, the weighted model consistently provided the highest accuracies of 97.80 ± 4.50% and 98.90 ± 1.50% for LOSO and 5-fold, respectively. As shown in Table 1, all other performance metrics followed the same trend. Therefore, it can be concluded that assigning weights to each class is the best I3D model to classify the body positions at sleep. Another observation was that not only did the average performance drop in LOSO compared to 5-fold, but all models also obtained higher standard deviations in LOSO, which could represent the differences in each subject’s data.

**Figure 3:**
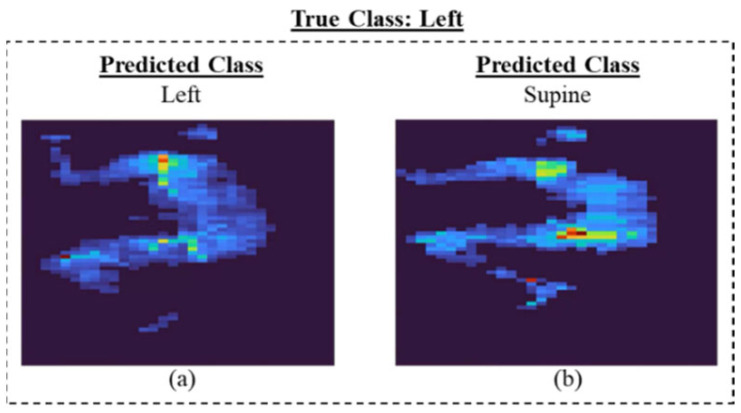
Sample video frames of I3D model for (**a**) correct and (**b**)incorrect classification.

Figure 2 shows that, in most of the models, the left side class was more misclassified than the right side. The supine class typically provided the lowest misclassification rate. Figure 3 displays sample frames of two misclassified videos. From this figure, it seems that the algorithm classified positions based on where most of the subject’s body was located. For example, when the participant lied on their left side (true class) but centered themselves in the middle of the mat as in Figure 3b, the model predicted this position as supine.

**Figure 4:**
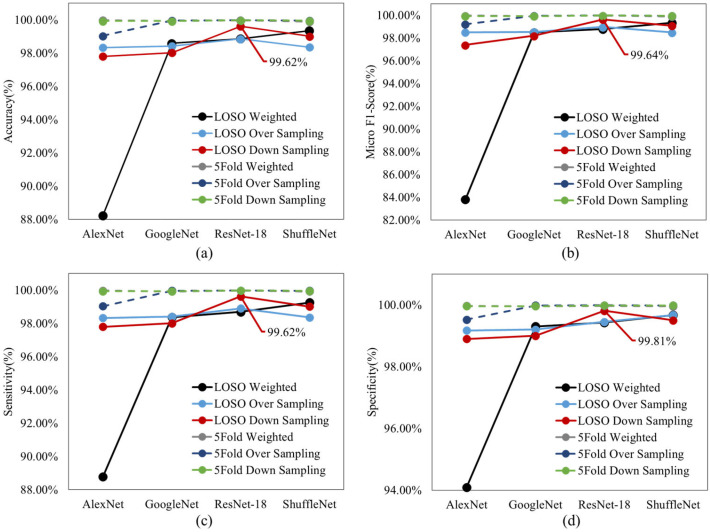
(**a**) Accuracy, (**b**) micro F1 score, (**c**) sensitivity, and (**d**) specificity of all four 2D pre-trained models with both LOSO and 5–fold validations.

**Figure 5:**
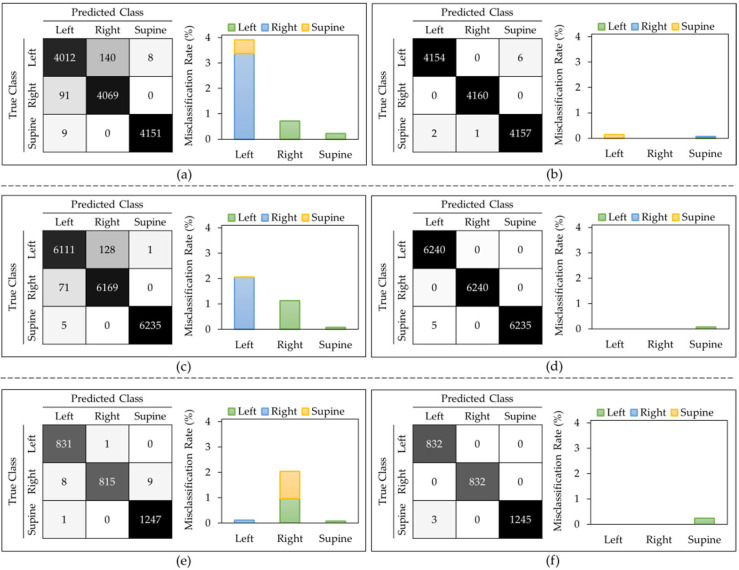
Best 2D models: Confusion matrix and misclassification rates of all 5 down-sampling runs for ResNet-18 (**a**) LOSO and (**b**) 5-fold. Confusion matrix and misclassification rates of all 5 over-sampling runs for ResNet-18 (**c**) LOSO and (**d**) 5-fold. Confusion matrix and misclassification rates of class weights model for ShuffleNet (**e**) LOSO and (**f**) 5-fold.

For the four pre-trained models, classification metrics of different models with different hyper-parameters were calculated, and the best models for LOSO and 5-fold cross validations were selected. All hyper-parameters were kept the same as the I3D model for a fair comparison. Figure 4 displays the classification metrics for all four pre-trained models for both LOSO and 5-fold. ResNet-18 model provided the highest overall performance with the down-sampling method. As expected, the performance of the models dropped slightly with LOSO compared to 5-fold validation regardless of the type of the model. Figure 5 shows the Confusion Matrices and Misclassification rates of the best pre-trained 2D models for five down sampling (832 images per class), five over sampling (1248 images per class), and for the class weight model (832 images per right-side and left-side classes, 1248 images per supine class) with both LOSO and 5-fold cross validation. The best down-sampled and over-sampled models are with ResNet-18, and the best-weighted models are with ShuffleNet. Overall, the ResNet-18 model received the highest accuracy when predicting the correct postures using the down-sampling method, with an accuracy of 99.62 ± 0.37% for LOSO and 99.97 ± 0.03% for 5-fold shown in Figure 4a.

Figure 5 displays that in all the LOSO models, every class had some misclassification associated with it, with the left class typically having the highest misclassification rate and the supine class having the lowest misclassification rate. However, in the 5-fold models, the right-side class never got misclassified, but the supine class always had some misclassification, with a maximum rate of 0.08%.

**Figure 6:**
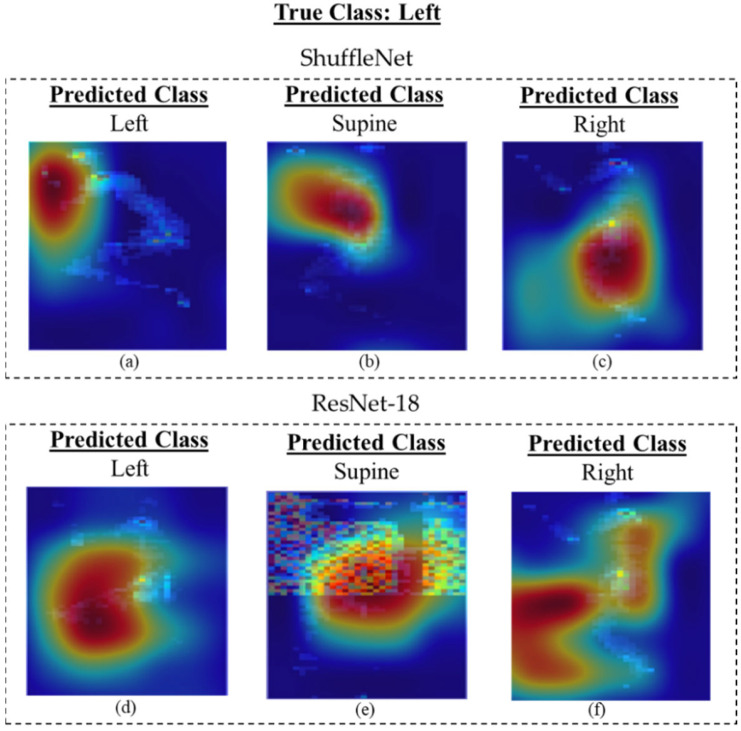
Grad-CAM images of the ShuffleNet and ResNet-18 models predicting left side posture: (**a**) correct prediction, (**b**) misclassified label as supine, and (**c**) misclassified label as right side with ShuffleNet. (**d**) Correct prediction, (**e**) misclassified label as supine, and (**f**) misclassified label as right side with ResNet-18.

To further evaluate the best-achieved models, ResNet18 and ShuffleNet, a gradient-weighted class activation map (Grad-CAM) was used to highlight the important regions of the image for model prediction. Grad-CAM allows a better understanding of which regions the models pay more attention to, in order to classify the images. For the ShuffleNet model, typically, misclassification occurred when the model was looking at regions of the image that did not align with the location of the body in that position. For example, in Figure 6b, it seems that the model was more focused on the center of the mattress, which is a correct focus for the supine class, or for Figure 6c, where the body was inclined to the right side of the mat. For the ResNet-18 model, the misclassification occurred when the bulk of the body was not found in the region of interest. For example, in Figure 6f, the model was primarily looking for the posture to be located toward the left side of the image; however, most of the posture was found towards the middle right of the image.

**Figure 7:**
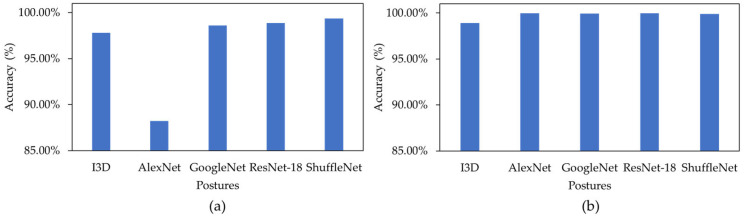
(**a**) A comparison between all weighted LOSO models, and (**b**) a comparison between all weighted 5-fold models.

All 2D weighted models provided approximately 1–2% higher accuracies than the I3D model when looking at the 5-fold cross validation. Notably, in order to compare the 2D and the 3D algorithms, the exact frames present in the 3D data were extracted as single frame inputs for the 2D CNN models. This led to some images being very similar to each other in the dataset. Therefore, it is more useful to compare the LOSO results of the 2D and 3D models, as there is no chance of data leakage. The ShuffleNet, ResNet-18, and GoogleNet received approximately 1–2% higher accuracies for the weighted method than the I3D model when looking at LOSO cross validation shown in Figure 7a.

There could be reasons to explain why these 2D models received a slightly higher accuracy when classifying this dataset. Firstly, for the 2D models, the dataset used to train, validate, and test was significantly larger than the I3D model, because the 2D models used frames from each video (16 frames per video), whereas the I3D model just looked at a video in its entirety. Therefore, the training and validation set were larger for the 2D models, allowing the model to have more information to learn from. Secondly, when testing the 2D models, only certain frames were misclassified, not an entire sequence of frames from a single video, whereas if there were specific frames within a video that made the I3D model misclassify the video during testing, then the entire video would be misclassified. When compared with previous work that was presented in the introduction, it can be seen that our proposed algorithms achieved higher performance, creating improved models for classifying the three postures. This could be due to a variety of reasons, such as an increase in the number of subjects, hyper-parameter tuning, and the pre-trained models.

Although these algorithms achieved high scores for distinguishing between three in-bed sleeping postures, it is important to recognize some limitations, as well. A limitation of the dataset was that only three postures were considered, and the prone position, another common sleeping posture, was not considered. Many papers chose to exclude the prone position as it often got confused with the supine position. Therefore, if the prone position was considered, then it can be assumed that the model would receive lower accuracies. Additionally, the data collected for this experiment were collected on healthy participants, with no history of pressure injuries, an age between 19 and 34, a height between 169 and 186 cm, and a weight between 63 and 100 kg. Therefore, this study may not replicate the population group that is most likely to use a pressure mat for pressure injury monitoring. Furthermore, the data collected in this study were set sleeping positions for 2 min, which is not a realistic use case for this system. In the future, it would be more beneficial to collect data in an overnight study, where the participants can lay in their normal sleeping postures, to train a model on more realistic positions. It should also be noted that within each class, there were different sub-classes associated with sub-positions that were not considered within the models of this study. However, we plan to consider that as one of our future works. Therefore, a more diverse dataset is required that includes the prone position, with participants within the aging population, in an overnight study.

Table 2 compares the previous studies that have used smart mats to classify three main in-bed postures using various algorithms and cross validation techniques. As depicted in Table 2, all previous studies validated their models by either k-fold or hold-out techniques despite the fact that the final models are expected to be used to predict the behavior of separate individuals; thus, it is important to use a subject-independent validation technique such as LOSO. Although the proposed model in [19] achieved the highest accuracy value than all other previous studies within this paper, this might not be a fair comparison as different studies used various validation techniques and datasets. For example, the results captured from the hold-out method will be directly affected by the type and quality of the data (50%) that were used for testing the model. Comparing the k-fold results, our proposed model provided the best performance in both 2D and 3D networks.

## 4. Conclusions

An Inflated 3D model was used to differentiate in-bed sleeping posture videos of the supine, right-side, and left-side classes. The 5-fold and leave-one-subject-out cross validation methods were used to evaluate the models. Due to the imbalanced dataset, down sampling, over sampling, and weighted models were used for each cross validation method to determine the best method to represent the data. The weighted model achieved the highest accuracy values of 98.90 ± 1.05% and 97.80 ± 2.14% for 5-fold and LOSO, respectively. To compare the Inflated 3D model, four 2D pre-trained deep learning models were used: AlexNet, ResNet-18, GoogleNet, and ShuffleNet. ResNet-18 achieved the highest accuracy of 99.62 ± 0.37% for LOSO, for the down sampling model. However, for a more accurate comparison of the weighted models, the ShuffleNet algorithm received the highest accuracies of 99.35 ± 0.43% for LOSO. Though these models provide a great basis for classifying various postures, there are many gaps within the research that need to be addressed, primarily with regard to the database, which can then be used for a more in-depth analysis. Some aspects include (1) collecting data on participants in various settings, such as at home, in hospitals, and in long-term care facilities, to have a more accurate representation of realistic data; (2) collecting data on various mattress stiffness, to evaluate the efficacy of a smart mat and ensure that the data being collected are constant throughout various environments; (3) adding common external devices, such as blankets and pillows, to provide a more realistic database; and (4) collecting overnight data to have a more holistic dataset that includes non-standardized positions as well as the transition between positions. This will open the possibility for a better analysis that will create models to be used in practice. Eventually, these models can be used as a preventative measure for pressure injuries as well as a monitoring tool for sleep quality. They can be used to either notify caregivers when it is time to change the position of the patients or can be used to monitor overnight sleeping to determine how much a person is moving in their sleep, thus evaluating the sleep quality of the person.

## Figures and Tables

**Table 1:** Performance metrics for I3D model.

	LOSO			5-FOLD		
	Down Sample	Over Sample	Weighted	Down Sample	Over Sample	Weighted
Accuracy (%)	92.05 ± 6.13	90.77 ± 7.52	97.80 ± 2.14	96.26 ± 3.71	97.28 ± 2.60	98.90 ± 1.05
F1-Score (%)	92.82 ± 5.62	91.63 ± 6.73	97.77 ± 2.21	96.43 ± 3.60	97.38 ± 2.50	98.86 ± 1.06
Sensitivity (%)	92.05 ± 6.13	90.77 ± 7.52	97.44 ± 2.25	96.32 ± 3.66	97.26 ± 2.66	98.73 ± 1.04
Specificity (%)	96.02 ± 3.06	95.38 ± 3.76	98.90 ± 1.05	98.13 ± 1.86	98.64 ± 1.30	99.43 ± 0.09

**Table 2:** A comparison between previous literature on classification of three in-bed postures: supine, right side, and left side.

Ref.	# Subs	# Postures	Algorithm	Cross Validation Method	Performance
[19]	19	3	DNN ^1^	Hold-out 50% for Testing	ACC ^2^: 99.70%
**[20]**	NA	3	SVM ^3^	10-fold	ACC: 94.14%
[9]	9	3	KNN ^4^	Hold-out 30% for Testing	ACC: 98.40%
[10]	3	3	HMM ^5^	10-fold	ACC: 90.40%
**Proposed Models**	**13**	**3**	**I3D DNN**	**5-fold**	**ACC: 98.90%**
				**LOSO**	**ACC: 97.80%**
			**2D DNN**	**5-fold**	**ACC: 99.97%**
				**LOSO**	**ACC: 99.62%**

^1^ DNN: deep neural network, ^2^ ACC: accuracy, ^3^ SVM: support vector machine, ^4^ KNN: k-nearest neighbour, ^5^ HMM: hidden Markov model.

## Data Availability

Not Applicable.
